# Combined Pharmacophore Modeling, Docking, and 3D-QSAR Studies of PLK1 Inhibitors

**DOI:** 10.3390/ijms12128713

**Published:** 2011-12-01

**Authors:** Shuai Lu, Hai-Chun Liu, Ya-Dong Chen, Hao-Liang Yuan, Shan-Liang Sun, Yi-Ping Gao, Pei Yang, Liang Zhang, Tao Lu

**Affiliations:** 1 Laboratory of Molecular Design and Drug Discovery, China Pharmaceutical University, Nanjing 211169, China; E-Mails: luai071@163.com (S.L.); tuna888@126.com (H.-C.L.); 0444909yuan@gmail.com (H.-L.Y.); 2 Department of Organic Chemistry, China Pharmaceutical University, Nanjing 211169, China; E-Mails: ssl870620@126.com (S.-L.S.); aladdincool@126.com (Y.-P.G.); scramble_Ant@163.com (L.Z.); 3 School of Traditional Chinese Pharmacy, China Pharmaceutical University, Nanjing 211169, China; E-Mail: peiyang1990@gmail.com; 4 State Key Laboratory of Natural Medicines, China Pharmaceutical University, Nanjing 211169, China

**Keywords:** PLK1, 3D-QSAR, pharmacophore, molecular docking

## Abstract

Polo-like kinase 1, an important enzyme with diverse biological actions in cell mitosis, is a promising target for developing novel anticancer drugs. A combined molecular docking, structure-based pharmacophore modeling and three-dimensional quantitative structure-activity relationship (3D-QSAR) study was performed on a set of 4,5-dihydro-1*H*-pyrazolo[4,3-*h*]quinazoline derivatives as PLK1 inhibitors. The common substructure, molecular docking and pharmacophore-based alignment were used to develop different 3D-QSAR models. The comparative molecular field analysis (CoMFA) and comparative molecule similarity indices analysis (CoMSIA) models gave statistically significant results. These models showed good *q*^2^ and *r*^2^ _pred_ values and revealed a good response to test set validation. All of the structural insights obtained from the 3D-QSAR contour maps are consistent with the available crystal structure of PLK1. The contour maps obtained from the 3D-QSAR models in combination with the structure based pharmacophore model help to better interpret the structure-activity relationship. These satisfactory results may aid the design of novel PLK1 inhibitors. This is the first report on 3D-QSAR study of PLK1 inhibitors.

## 1. Introduction

Polo-like kinases (PLKs), a family of serine/threonine protein kinases, have attracted much attention as important elements that regulate cell cycle progression, particularly mitosis. In homo sapiens, four PLK homologs have been identified (PLK1, PLK2, PLK3 and PLK4) [1], and more recently, PLK5 has been identified. However, PLK5 is short of a kinase domain and may not function in cell cycle regulation [2]. They all share a highly conserved *N*-terminal catalytic kinase domain and a *C*-terminal region composed of “polo boxes” (only one in PLK4). PLK1 is the most investigated member of the family and has been widely considered as an anticancer target [3–5]. PLK1 is expressed primarily in dividing cells, which functions in mitosis entry, centrosome maturation, kinetochore assembly, bipolar spindle formation, cytokinesis and the exit of mitosis [6–13]. Knockdown or pharmacologic inhibition of PLK1 in tumor cells results in defects in centrosome maturation and separation, mitotic spindle formation and chromosome alignment, leading to disruption of cell mitosis and even apoptosis [14–16].

PLK1 is strongly associated with human malignancy due to its frequent over-expression in a variety of tumors with poor prognosis, such as breast cancer, ovarian cancer, pancreatic cancer, lung cancer, endometrial cancer, head and neck cancer, gastric cancer, prostatic cancer, *etc*. [4,17]. Given the oncogenic amplification and transforming potential of PLK1, there is a high level of interest and an increasing effort to inhibit its enzymatic activity with small-molecule compounds to the catalytic domain (ATP-binding site) for cancer therapy. Currently seven PLK1 inhibitors are in clinical trials and well tolerated in humans [18–20]. Recently, the 4,5-dihydro-1*H*-pyrazolo[4,3-*h*]quinazoline derivatives were reported as a novel class of PLK1 inhibitors, showing high potency at a nanomolar level [18,20,21]. Due to favorable biochemical profiles, high potency both *in vivo* and *in vitro*, and the acceptable oral bioavailability, two compounds of this class were subjected to clinical trials [18,20]. However, the study of type II PLK1 inhibitors is relatively slow. Only one case of potent type II inhibitors was reported by Keppner and coworkers in 2009 [22].

To date, there have not been any reports on 3D-QSAR studies of PLK1 inhibitors. Herein, we report the application of pharmacophore modeling, docking, comparative molecular field analysis (CoMFA) [23] and comparative molecular similarity analysis (CoMSIA) [24] 3D-QSAR methods to the 4,5-dihydro-1*H*-pyrazolo[4,3-*h*]quinazoline derivatives. This study was undertaken to gain insights into molecular mechanisms and structural requirements crucial for potential inhibition of PLK1, which could be useful in the design of novel PLK1 inhibitors. The CoMFA and CoMSIA analyses were conducted to investigate how the activity is influenced by steric, electrostatic, hydrophobic, and hydrogen bonding interactions. The 3D-QSAR models obtained from both the ligand- and structure-based alignments were both found to be statistically valid in terms of the interpretation of interaction mode and the predictability to internal and external compounds. The contour plots obtained from the 3D-QSAR models correlated well, not only with the detailed interactions between the ligands and active-site residues in the crystal structures of PLK1, but also the pharmacophore features directly derived from the receptor-ligand interactions in crystal structures. The developed computational models are expected to help with better understanding of the QSAR of this class of compounds, as well as ensuring the researcher an in-depth analysis about the lead compounds for PLK1 inhibitor in further studies. To the best of our knowledge, this work will be the first 3D-QSAR study of PLK1 inhibitors reported.

## 2. Results and Discussion

### 2.1. Multiple Structure Alignment Analyses

**Figure 1:**
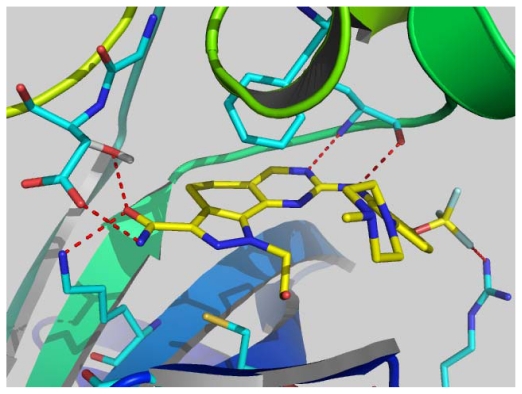
The co-crystal binding mode of compound **73** with PLK1. The hydrogen bond is represented with red dotted line.

The accuracy and reliability of the CoMFA and CoMSIA model is directly dependent on the structural alignment rule. Therefore, before PLS analyses to construct the 3D-QSAR models, we performed the structure and ligand based alignment to find the effective alignment to this dataset (Table 1). Because the alignments involved were actually based on the co-crystal structure of compound **73** with PLK1, a preliminary analysis on its binding mode was necessary. Figure 1 shows the co-crystal interaction mode of compound **73** with PLK1 (2YAC, resolution: 2.2 Å). The core of compound **73** is sandwiched between Phe183 at the bottom of the ATP binding pocket and Cys67 in the back of the G-loop. An aromatic ring stacking interaction is found between the Phe183 and compound **73**, which has an important influence on the conformational equilibrium of the whole compound. The 4-methylpiperazinyl moiety penetrates to the solvent accessible region, which may be involved in hydrophilic interactions. The 2-hydroxyethyl group positions at the same place related to the ribose moiety of ATP, which is a site tolerant to the long chain substituent. In addition to two conservative hydrogen bonds formed with the hinge region residue Cys133, the amide moiety and the trifluoromethoxyl group are engaged in three and one hydrogen bonding interactions, respectively. The knowledge on binding mode will assist in the evaluation of compound alignments as well as QSAR analyses.

**Figure 2:**
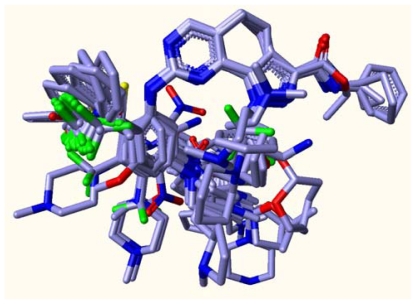
The common substructure based alignment.

**Figure 3:**
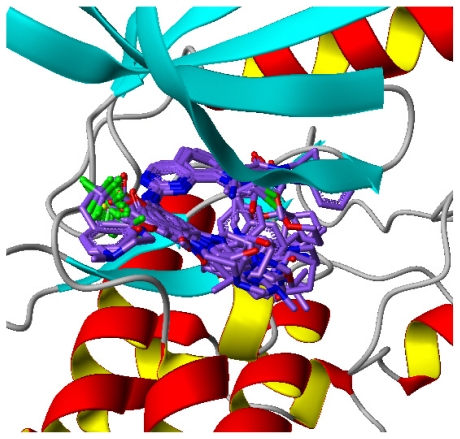
The resultant conformations from GLIDE docking.

**Figure 4:**
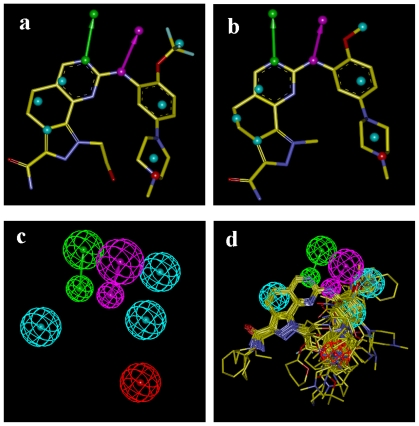
(**a**) Pharmacophore model derived from 2YAC; (**b**) Pharmacophore model derived from 3KB7; (**c**) The merged model; (**d**) The compounds alignment based on the merged model. Features are color-coded with magenta for hydrogen-bond donor, green for hydrogen-bond acceptor, light-blue for hydrophobic, red for ionizable positive.

As depicted in Figure 2, all 73 compounds were aligned well, using the common substructure based method. GLIDE performed quite well, as most conformations bind in a way analogous to the bound ligand of 2YAC, *i.e.*, compound **73** (Figure 3). Thus, the alignment derived by GLIDE docking is considered reasonable. Figure 4a,b illustrate two pharmacophore models deduced from the PLK1 crystal structures 2YAC and 3KB7 by LigandScout. It is obvious that they share nearly identical features. To cover the most common features that may be required by PLK1 inhibitory potency, we clustered and subsequently merged them to a new pharmacophore model. This merged model (Figure 4c) consists of one hydrogen-bond acceptor, one hydrogen-bond donor, three hydrophobic and one ionizable positive, which is simplified by discarding three redundant hydrophobic features. Figure 4d shows the result of pharmacophore mapping of those compounds, which also suggests an excellent alignment.

Taken together, the results from common substructure and the merged pharmacophore based alignments, as well as GLIDE docking, proved to be reasonable and effective. It was difficult to judge which alignment would be more practicable, therefore, they were all subjected to the next step for model generation to further investigate their applicability and gain a more extensive insight to QSAR of pyrazoloquinazoline PLK1 inhibitors.

### 2.2. CoMFA and CoMSIA Statistical Results

**Figure 5:**
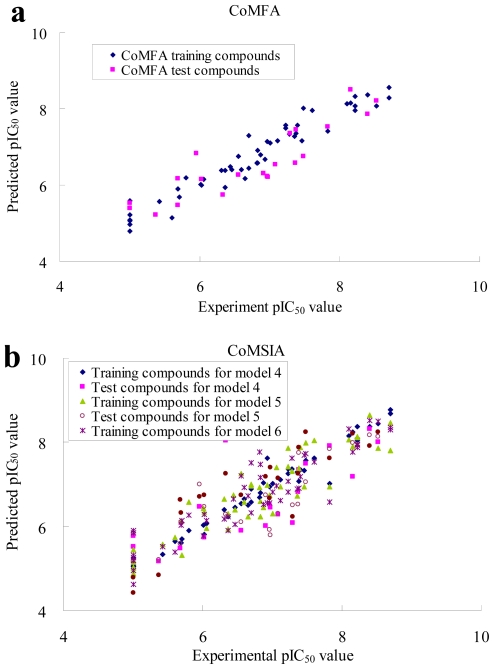
Plot of predicted *vs.* experimental values of (**a**) CoMFA model 2 and (**b**) CoMSIA models 4, 5 and 6.

Owing to the fact that common substructure, GLIDE docking and the pharmacophore methods all produced acceptable alignments of 73 known PLK1 inhibitors, the corresponding CoMFA and CoMSIA analyses were performed independently for further comparison. The statistical results of PLS analyses for CoMFA and CoMSIA studies are listed in Table 2. The pharmacophore based model yielded *q*^2^ = 0.628 and *r*^2^ = 0.941 for CoMFA, whereas the GLIDE docking and common substructure based model produced a lower *q*^2^ value of 0.283 and 0.578, and *r*^2^ value of 0.420 and 0.867 for CoMFA, respectively. Multiple CoMSIA models were derived based on three types of alignment, with various combinations of steric, electrostatic, hydrophobic, hydrogen bond donor and hydrogen bond acceptor fields. To get a clear view, only parameters of models whose *q*^2^ value are higher than those of other models derived from the same alignment were considered. It is obvious that CoMSIA models from common substructure, GLIDE docking and pharmacophore based alignments showed comparable results. The inhibitory activities (pIC_50_), the predicted activities using the CoMFA and CoMSIA models, and the corresponding residual values for the training set compounds are listed in Tables 3 and 4. Graphic representations of experimental *vs.* predicted inhibitory activity of training set for pharmacophore-based CoMFA and typical CoMSIA models are shown in Figure 5. In all, the CoMFA and CoMSIA models we constructed possess high *q*^2^ and *r*^2^ value, indicating that they have good internal predictive ability and that results were not based on any chance correlation. To validate both the predictability and accuracy of the models for external compounds, the predictive correlation coefficient *r*^2^ _pred_ was calculated for the test set. As shown in Tables 3 and 4, the *r*^2^ _pred_ value of pharmacophore-based CoMFA model and CoMSIA models spans from 0.605 to 0.827 and most of the residual values are less than 1.0, revealing that the models are highly reliable and can be used to predict the biological activities of novel compounds; whereas, the *r*^2^ _pred_ value of CoMFA models from GLIDE docking and common substructure based alignment is somehow lower, reflecting poor predictive ability. The plots of experimental *vs.* predicted inhibitory activity of test set for CoMFA and CoMSIA models are shown in Figure 5, showing that the predicted activities were in good agreement with the original data and the reliable CoMFA and CoMSIA models have a robust external predictive ability.

It can be concluded easily that the best model for CoMFA was obtained from the pharmacophore-based method (model 2) while it was difficult to distinguish the best CoMSIA model because there is no significant difference between PLS statistical results. Hence, we have paid attention to all CoMSIA models (models 4, 5 and 6), considering the representation of different fields, the satisfactory internal and external predictive ability in terms of *q*^2^ and *r*^2^ _pred_ value, respectively.

### 2.3. CoMFA Contour Maps

The results of CoMFA analyses from pharmacophore-based alignment (model 2) are displayed as color-coded contours, allowing visual inspection of regions responsible for favorable or unfavorable interactions with PLK1. The green contours indicate regions where bulky substitution enhances binding affinity, and the yellow contours suggest regions where bulky substitution reduces the binding affinity. In the electrostatic interaction map, the blue contours indicate regions where more positively charged substituents are favored and the red contours suggest regions where more negatively charged substituents are favored. The favorable and unfavorable contributions of both fields were plotted as default proportion (80:20). Since the QSAR models were developed based on the information from receptor (docking, structure-based pharmacophore and common substructure from crystal bound conformation), the contour maps produced by CoMFA and CoMSIA could be superimposed onto the PLK1 structure. Thus, to get a straightforward insight into the steric and electrostatic interaction between compounds and PLK1, we introduced the van der waals surface or electrostatic potential surface of protein as background.

**Figure 6:**
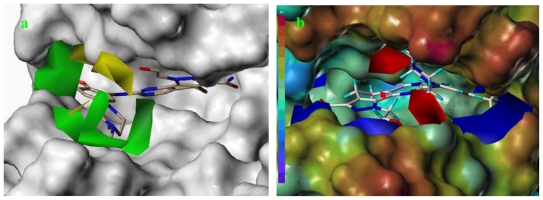
The CoMFA contour map of model 2 combined with compound **73**. (**a**) Steric field distribution on the background of protein surface; and (**b**) electrostatic field distribution on the background of electrostatic potential surface colored from purple to red owing to the increase of electron density. Green contours indicate regions where bulky groups increase activity, whereas yellow contours indicate regions where bulky groups decrease activity. Red contours suggest negative charge favoring activity, whereas blue contours suggest positive charge favoring activity.

The steric and electrostatic fields contribute to 61.9% and 38.1% of the variance, respectively. The steric contour map is shown in Figure 6a with one of the most potent inhibitors, *i.e.*, compound **73** as a reference. A moderate green contour is seen in proximity to the *o*-trifluoromethoxyl group of phenyl ring, but sandwiched by the protein surface, suggesting that only the medium-sized substituent is favored at this position such as methoxyl, trifluoromethoxyl, and methyl. The large green contours are found around the 4-methylpiperazinyl moiety at 5′ position of the phenyl ring, which penetrate to the solvent accessible region. This indicates that diverse substituents with bulky size at 5′ position of the phenyl ring are favorable to activity and their orientations are tolerable in space, except those extending to the upper side. Those situations for steric favorable substituents are the same for compounds **35**, **42**, **44**, **47**, **48**, **51**, **53**, **58** and **59**, all of which show better activity (below 10 nM) and have moderate and bulky groups at 3′ and 5′ positions of the phenyl ring. However, compounds that have only one steric favorable site show only moderate binding affinity, e.g., compounds **5**, **12**, **14**, **15**, **22**, **25**, **28**, **29**, **38** and **33**, demonstrating that both sites are indispensable. The emergence of yellow contours in the front of 2-hydroxyethyl group suggests that a more bulky substituent at position 1 of pyrazole ring would lower the activity. For example, replacement of 2-hydroxyethyl group with longer substituents (compounds **69**, **70** and **72**) led to a significant reduction of potency. Another yellow contour over the phenyl ring shows that a bulky substituent in this area is not favorable; but when the phenyl ring is flipped vertically by ~90 °, this area can also be occupied by substituents, such as compounds **11**, **16**, **21**, **27**, **30** and so on. In all, there is a definite requirement of an appropriate shape to exhibit high potency when designing novel PLK1 inhibitors, and thus it is important to pay attention to the steric characteristics.

Electrostatic contour maps are also shown with compound **73** as a reference (Figure 6b). In general, red contour maps are close to heteroatoms such as nitrogen and oxygen, whose partial atomic charges are highly negative. Two main red contours are found close to 4-methylpiperazinyl moiety, of which N atoms bear negative charges, indicating negative potential is preferred in these areas. This trend can be reflected in the activities of compounds **35**, **44**, **47**, **48**, **49**, **51**, **52**, **53**, **58**, **59** and **73**, which all have tertiary amine substructures. Confusingly, the simple replacements of 4-methylpiperazinyl moiety with other tertiary amines for compound **53**, either open-chain or cyclic, result in decreased potency against PLK1 from 10-fold to more than 300-fold, for compounds **40**, **41**, **43**, **56**, **57**, **60**, **61**, **62**, **63**, **64**, **65** and **68**. This can be explained by taking the steric factor into account, that an unfavorable steric contour exists over the 4-methylpiperazinyl moiety as illustrated in Figure 6a. With respect to the favorable positive potential, the blue contours are distributed around the *o*-trifluoromethoxyl group, 2-hydroxyethyl group and amide moiety, where the negative charge of protein surface is concentrated, suggesting that positive charged groups such as substituents with electron-withdrawing atoms increase the activity compared to the hydrogen atom. This is consistent with the increase of potency for compound **53** as compared to compound **35**, due to the replacement of hydrogen atom with fluorine atom. A similar situation can be observed between compounds **71** and **73**, for which the replacement of nitrogen atom with oxygen atom leads to increased activity. Besides, two small blue regions close to the amide moiety also represent the preferred electrostatic interaction around there, indicating that decreasing the electron-withdrawing effect would cause a reduction of binding affinity, such as compounds **1**, **2**, **3**, **4** and **36**. Collectively, the distribution of electrostatic contours is well corroborated with the electrostatic potential information of the PLK1 active site, demonstrating its rationality.

### 2.4. CoMSIA Contour Maps

**Figure 7:**
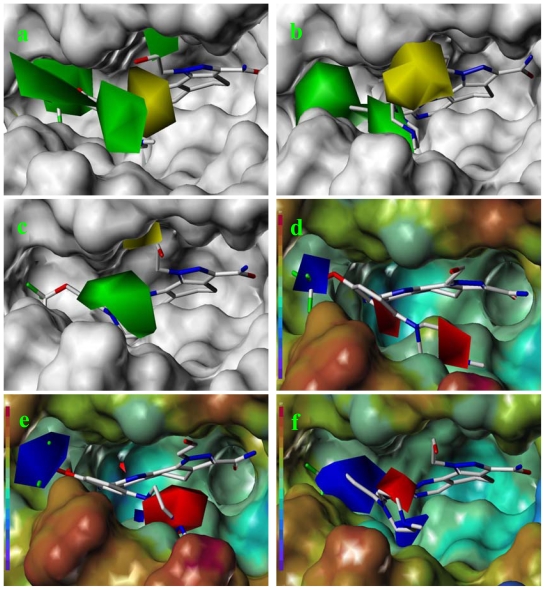

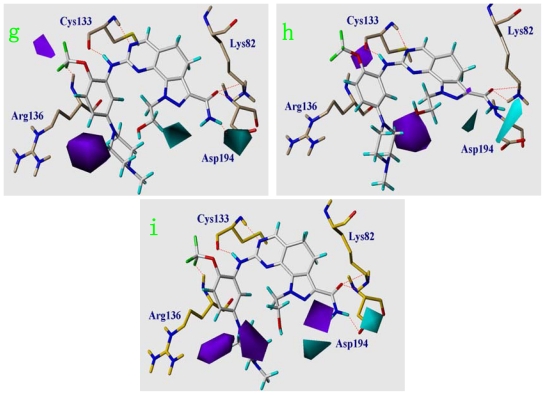
The CoMSIA contour map combined with compound **73**. Steric field distribution for (**a**) model 4, (**b**) model 5 and (**c**) model 6, on the background of protein surface. Electrostatic field distribution for (**d**) model 4, (**e**) model 5, and (**f**) model 6, on the background of electrostatic potential surface colored from purple to red owing to the increase of electron density. Hydrogen bond donor field distribution for (**g**) model 4, (**h**) model 5 and (**i**) model 6. Green contours indicate regions where bulky groups increase activity, whereas yellow contours indicate regions where bulky groups decrease activity. Positive potential favored areas are in blue, and positive potential unfavored areas are in red. Cyan and purple contours indicate favorable and unfavorable hydrogen bond donor group. The hydrogen bond is represented with orange dotted line.

The CoMSIA contour maps of four models (models 4, 5 and 6), based on different alignment methods or combinations of various fields, are shown in Figure 7, which are depicted with compound **73** as the default from nearly the same viewpoint for a convenient comparison and analysis. The steric and electrostatic contours are colored identically with CoMFA contours map. In addition, the hydrophobic interactions are shown by yellow and white contours, whereas hydrogen bond donor interactions are represented by cyan and purple contours, indicating their favorable and unfavorable regions. The contributions of fields to the variance are listed in Table 2. It is evidenced that the electrostatic field contributes about 1.7–3 times more than the steric field, which is the opposite of corresponding relationships in the CoMFA models. As the alignment results are identical for CoMFA and CoMSIA models, this discrepancy may be explained by the different implementations of the fields for CoMFA and CoMSIA. Thus, we assume that both steric and electrostatic fields play important roles in the binding affinity and should be given equal attention.

Since the steric and electrostatic interactions have been discussed above in detail, a critical eye has been given to the comparison of the contour distributions. As shown in Figure 7a–c, the green contours mainly concentrate around the 4-methylpiperazinyl moiety and the trifluoromethoxyl group, denoting that the bulky substituents are indeed favorable to these regions; a large yellow contour is constantly located between the 2-hydroxyethyl group and 4-methylpiperazinyl moiety, suggesting a potential steric clash may exist between those two substituents. However, there is a distinct difference in the steric contour above the 2-hydroxyethyl group as the green contour in model 4 is conversely yellow in model 6. It can be found that a small sub-pocket is positioned over the 2-hydroxyethyl group, which means bulky substituents are not acceptable, such as 2-(tetrahydro-2*H*-pyran-2-yloxy)-ethyl and 3-aminopropyl groups, leading to lower binding affinities for compounds **70** and **72**, respectively. Therefore, we conclude that a small yellow contour is more appropriate in that position. As for the electrostatic field, it can be observed that the contours of CoMSIA models are more concentrated around the 2-hydroxyethyl group and 4-methylpiperazinyl moiety (Figures 7d–f) in comparison with those of CoMFA model. In spite of this, common characteristics still exist. Commensurate with the CoMFA model, in all three CoMSIA models, a blue contour and large red contour are close to the trifluoromethoxyl group and the 4-methylpiperazinyl moiety, respectively, denoting the electrostatic nature of those two positions are authentically reflected. In addition, a middle-sized blue contour is also proximate to 4-methylpiperazinyl moiety in the CoMSIA model derived from common substructure based method (model 6). Since the most frequently used substituents at position 5′ of phenyl ring are various tertiary amines, the blue contour may account for the electron-deficient methylenes. Therefore, the substituents at position 5′ of the phenyl ring should consist of the electron-withdrawing atom and the electron-deficient atom simultaneously rather than solely the electron-withdrawing atom, such as carbamoyl or sulfoamino group. This conclusion is supported by the fact that the position 5′ of phenyl ring is oriented to the solvent accessible region of PLK1 and the substituents with the ionizable groups at that position will be ionized in the solution, stabilizing interaction and enhancing potency.

Areas favored by hydrogen bond donors are shown in cyan and magenta, respectively (Figures 7g–i). For all three CoMSIA models, two cyan contours are equidistant and close to the amino group of the amide moiety, mirroring the potency of two hydrogen atoms in the NH_2_ group to form hydrogen bonds with the residues of receptor in the corresponding orientations, such as Asp194. These contours can be associated with the increment in activity when the NH group of the amide moiety changes from ethoxyl and substituted amino groups in compounds **1**, **2**, **3**, **4** and **36**, implying the NH group plays a major role in binding to the PLK1 active sites. The purple contours are found in common around the 4-methylpiperazinyl moiety, denoting the disadvantage of the hydrogen bond donor at this position for activity. This is corroborated with the distribution of electrostatic contours. A small purple contour is shown near the trifluoromethoxyl group that formed a hydrogen bond with the backbone NH group of Arg136 in our models and the guanidine NH group of Arg57 in PLK1 crystal structure (2YAC), respectively. Thus, the hydrogen bond acceptors at this position are favorable. A confusing purple contour is observed near the NH group of the amide moiety. As a crystal structure, and our models confirm the favor of the hydrogen bond donor at this area, this contour cannot be associated to the NH group. From a systematic investigation of the conformations superimposed with contours, we found the substituents at position 1 of pyrazole of compounds **69** and **72** might account for that purple contour, whose hydrogen bond donor groups reach this point due to the flexibility of the alkyl chain.

### 2.5. Comparison of Pharmacophore Model and CoMSIA Model

**Figure 8:**
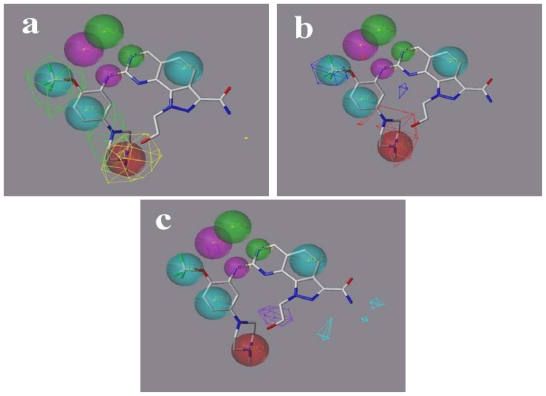
The pharmacophore model superimposed with (**a**) steric; (**b**) electrostatic and (**c**) hydrogen bond donor and acceptor contours of model 6. The pharmacophore features are colored the same as in Figure 4. The contours are depicted as mesh.

Considering that the pharmacophore model we have constructed also consists of hydrogen bond related features and a hydrophobic feature, it would be significant to compare it with the CoMSIA models. Hence, the merged pharmacophore model was reproduced using Unity module in SYBYL 6.9 [25]. The graphical interpretation of the superimposition of the features and contours reflecting the hydrophobic and hydrogen bond donor fields is shown in Figure 8 with compound **73** as a reference. The contours reflect the corresponding fields of model 6 (derived from pharmacophore-based alignment). Two large green contours are close to two hydrophobic features located at the trifluoromethoxyl group and in the vicinity of the phenyl ring, suggesting bulky and hydrophobic substituents at these positions are favorable (Figure 8a). The ionizable positive feature is covered by the large red contour, indicating a hydrophilic characteristic is preferred here (Figure 8b). The hydrogen bond donor and acceptor features do not intersect with the contours related to the hydrogen bond donor field (Figure 8c). Despite only partial pharmacophore features overlapping well with corresponding contours, it is still considered reasonable because the features not overlapped with contours belong to the maximum common substructure of compounds or are conservative in most compounds used in this study, while the CoMSIA method mainly focuses on the variable parts for a class of compounds. In this sense, our 3D-QSAR model and pharmacophore model complement each other well in elaborating the interaction mode of compound.

### 2.6. Structural Insights from 3D-QSAR and Pharmacophore Studies

**Figure 9:**
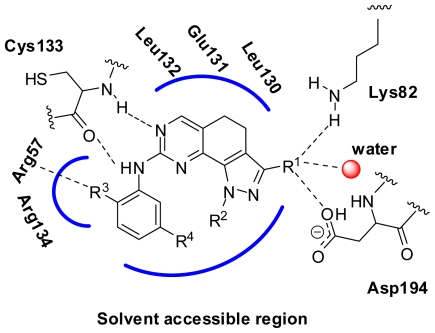
Schematic representation for the SAR of 4,5-dihydro-1*H*-pyrazolo[4,3-*h*]quinazoline derivatives as PLK1 inhibitors. R^1^: medium-sized substituent with hydrogen bond donor and acceptor; R^2^: open-chain alkyl group with less than three carbon atoms or unsubstituted hydrogen; R^3^: hydrophobic group with small size and strong electron-withdrawing atom, especially hydrogen bond acceptor; R^4^: bulky substituents simultaneously with hydrophobic and hydrophilic moiety.

Our analyses found that the electrostatic, steric and hydrogen bond donor characteristics are highly desirable for potent inhibitory activity. The contour maps show that a moderate bulky substituent with hydrogen bond donor at position 3 of pyrazole ring (R^1^), a moderately bulky and hydrophobic group with electron-withdrawing heteroatom at position 2′ of phenyl ring (R^3^), and a bulky and amphoteric substituent with both hydrophilic and hydrophobic moieties at position 5′ of phenyl ring (R^4^), can play important roles in enhancing binding affinity. Moreover, the length of alkyl chain for the substituent at position 1 of pyrazole ring (R^2^) should be no more than three carbon atoms. In addition, two hydrogen bonds formed between compounds and Cys133 in hinge region are important which can induce the conformation of the whole compound in the ATP pocket. Thus, influencing these two hydrogen bonds should be avoided when changing substituents at other positions. These insights are consistent with the structural features of ATP pocket, further indicating that our 3D-QSAR models are reasonable. As depicted in Figure 9, R^2^ and R^4^ groups project into the solvent accessible region, thus allowing a larger extent of variability for the steric, electrostatic and other properties of substituents. Nevertheless, the steric clashes between R^2^ and R^4^ groups should be avoided. Moreover, R^2^ and R^4^ groups still have an impact on PLK1 selectivity. The reasonable combinations of substituents at these two positions can increase the selectivity of PLK1 against PLK2-3 up to 5000 times. Although R^1^ group can form hydrogen bonds with a water molecule, substituents that discard this interaction may also be positive to the enhancement of binding affinity and target selectivity. This is concluded from the study of Fernandez and coworkers, which indicated that sculpting the shifting hydration patterns of the target would stabilize the protein surface and avoid disfavored induced fit [26].

## 3. Materials and Methods

### 3.1. Dataset

**Figure 10:**
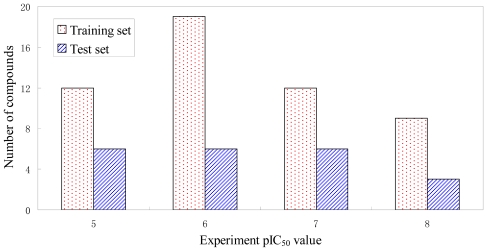
Distribution of activities (pIC50) for the training set and the test set *versus* numbers of compounds. The training set and the test set are colored as red and blue, respectively.

All compounds used in the present study were taken from the literature [18,20,21]. Of the 73 compounds, 52 ones (unasterisked in Table 1) were selected randomly as training set for model construction and the remaining 21 ones (asterisked in Table 1) were used as test set for model validation, according to biological activity range and structural diversity. The IC_50_ values of all compounds for PLK1 inhibition were normalized and converted to the logarithm unit of molar grade (pIC_50_ = −log IC_50_), which spanned 4 orders of magnitude (5.00–8.70). The distribution of activity data and the number of compounds were shown in Figure 10 to confirm with the test set as a true representative of the training set.

The X-ray crystal structures of this class of compounds bound with PDK1 are available from the protein data bank (PDB). The bound conformation of compound **73** (PDB code: 2YAC) [18] was used as a template to build the 3D structures for both training and test set compounds. The partial charge was calculated with Gasteiger-Hückel method. The common structure was constraint for each compound and only the varying parts were energy minimized by using conjugate gradient method and Tripos force field until an energy gradient of 0.05 kcal/mol was reached. These works were all done in SYBYL 6.9.

### 3.2. Conformational Alignment

Structure alignment is considered as an important and critical step in CoMFA and CoMSIA analyses because this affects the reliability of the models. In order to avoid bias towards a particular alignment method, the structure-based and ligand-based alignments were both used in this study. It should be noted that a study that specifically seeks to understand the influence of alignment methods on the predictive performance of 3D-QSAR model is an important direction but extended in the work presented here. Herein, the common substructure, molecular docking and pharmacophore-based alignment were performed to build the 3D-QSAR models. Meanwhile, the docking and pharmacophore studies would also provide beneficial insight into ligand-receptor interactions to help better understand the structure-activity relationship.

#### 3.2.1. Common Substructure Based Alignment

**Figure 11:**
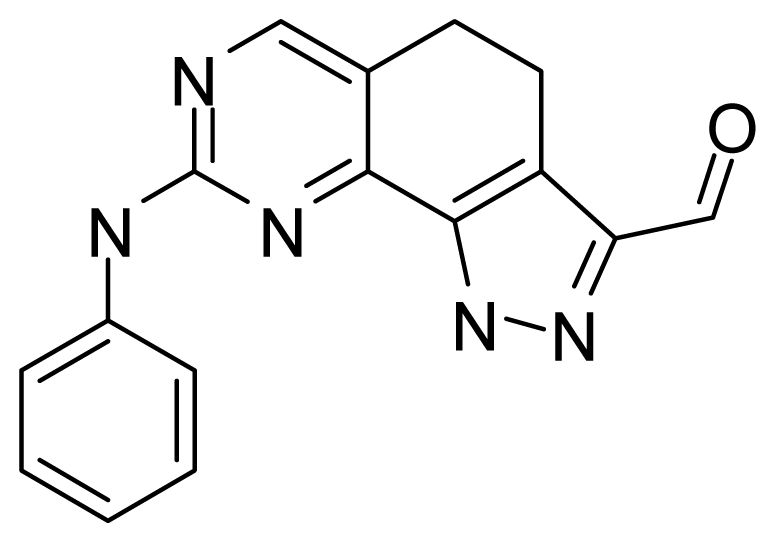
The most common substructure used in common substructure-based alignment.

The key assumptions of CoMFA and CoMSIA are that compounds with common substructure always adopt a similar conformation when binding with the target and the common substructure in each compound contributes equally. Therefore, we selected the co-crystal structure of compound **73** from 2YAC as the template to align the remaining compounds using the “align database” command in SYBYL 6.9. The common substructure used for the alignment is shown in Figure 11.

#### 3.2.2. Molecular Docking Based Alignment

Molecular docking was carried out to obtain reasonable molecular alignments for developing receptor-based 3D-QSAR models. At the beginning, we tested the applicability of three well-known docking software, *viz.* CDOCKER [27,28] in Discovery Studio 2.5, GOLD 5.0 [29,30] and GLIDE 4.5 [31,32] in Maestro 8.0, by checking if the conformation of the bound ligand in PLK1 crystal structure can be reproduced, and whether the common substructure of all compounds in both training and test sets can overlap well with each other in a way analogous to the bound ligand in PLK1 crystal structure. Docking conformations output by both CDOCKER and GOLD overlapped in a chaotic state, suggesting a failure of alignment. In contrast, GLIDE performed quite well. Thus, GLIDE was eventually selected as the docking tool.

The 3D structure of PLK1 (2YAC) in docking study was downloaded from Protein Data Bank. For GLIDE, the PDB structure was prepared using the “protein prepare wizard” automatically and subsequently its grid file was generated in Maestro 8.0. The initial conformation of compound used was obtained by conformational search in water with force filed of OPLS_2005 based on mixed torsional/low-mode sampling method in Maestro 8.0. The binding site was defined by the co-crystal ligand (compound **73**) itself for all three docking software. The XP mode (extra precision) was selected and post-docking minimization was conducted to penalize highly strained ligand geometries and eliminate poses with eclipsing interactions. Finally, other options not mentioned above were kept as default.

#### 3.2.3. Pharmacophore Based Alignment

The structure based pharmacophore model can be derived directly from ligand-protein co-crystal structure and thus can reflect more reliable combination of the essential features required for the relating biological potency [33]. As the compounds used in 3D-QSAR analyses belong to the same class and the co-crystal structures of PLK1 are available, the structure-based pharmacophore was generated utilizing LigandScout 2.02 [34], which is based on a sophisticated ligand-protein complex interpretation algorithm. Two PLK1-ligand co-crystal structures (2YAC and 3KB7) [18,20] available were chosen. When creating pharmacophore model, the “Phase” mode was selected with waters and other heteroatom ignored due to their non-conservation in crystal circumstance. This produced two pharmacophore models. Considering that pharmacophore should contain the most common features and these two models indeed share some identical features, we compared and clustered them in Discovery Studio 2.5 to draw a new pharmacophore model. This model was eventually used to align compounds in Discovery Studio 2.5, during which the conformations of compounds were generated with “best” option and the fitting method was “flexible” with the maximum omitted features of 3.

### 3.3. CoMFA and CoMSIA Methodology

The CoMFA and CoMSIA analyses were carried out with the RHEL 4.0 operating system using SYBYL 6.9. In CoMFA study, the aligned compounds were placed in the 3D cubic lattice with grid spacing of 2.0 Å. The standard CoMFA steric and electrostatic fields were calculated using a sp3 carbon atom as steric probe and a +1 charge as electrostatic probe, with Lennard-Jones potential and the Coulombic potential, respectively. The cut off value for both fields was set to 30 kcal/mol and the minimum-sigma (column filtering) was set to be 2.0 kcal/mol to reduce the noise by omitting those lattice points. The five fields of CoMSIA (steric, electrostatic, hydrophobic, hydrogen bond donor and acceptor) were calculated for each lattice with a grid of 2 Å by employing a common probe atom with 1 Å radius, +1 charge, and hydrophobic and hydrogen-bond property values of +1 [24]. The attenuation factor was set to the default value of 0.3 for the Gaussian function.

### 3.4. Partial Least Squared (PLS) Analyses and Validation

The relationship between the structures and the biological activities derived by the PLS algorithm. CoMFA and CoMSIA descriptors were used as independent variables and pIC_50_ values were used as dependent variables in PLS to generate corresponded 3D-QSAR model. The predictive ability of the models was evaluated by leave-one-out (LOO) algorithm, which gave the optimal number of component (ONC), the lowest standard error of prediction and cross-validation coefficient (*q*^2^), calculated with Equation 1,

(1) $$q^{2}=1-\frac{\sum {\left({\text{Y}}_{\text{pred}}-{\text{Y}}_{\text{exp}}\right)}^{2}}{\sum {\left({\text{Y}}_{\text{exp}}-{\text{Y}}_{\text{mean}}\right)}^{2}}$$

where Y_pred_, Y_exp_ and Y_mean_ are the values (pIC_50_) of the predicted activity, experimental activity and mean activity for compounds in training set, respectively.

The analysis of non-cross validation was performed to calculate the conventional *r*^2^ using the ONC obtained from the LOO analysis. Validation of the utility of the model as a predictive tool was carried out by predicting the activity of an external test set of 21 compounds. The predictive *r*^2^ (*r*^2^ _pred_), reflected the predictive power of the CoMFA and CoMSIA models, was calculated using Equation 2,

(2) $$\begin{array}{c} \text{PRESS}=\sum {\left({\text{Y}}_{\text{exp}}-{\text{Y}}_{\text{pred}}\right)}^{2}\\ \text{SD}=\sum {\left({\text{Y}}_{\text{exp}}-{\text{Y}}_{\text{mean}}\right)}^{2}\\ {r^{2}}_{\text{pred}}=1-\text{PRESS}/\text{SD} \end{array}$$

where SD is the sum of the squared deviations between the experimental activities of the compounds in the test set and the mean activity of the compounds in the training set, PRESS is the sum of the squared deviations between predicted and experimental activities for every compound in the test set.

## 4. Conclusions

The 4,5-dihydro-1*H*-pyrazolo[4,3-*h*]quinazoline derivatives are a class of novel, potent, selective and orally bioavailable PLK1 inhibitors with reasonable SAR and strong quantitative correlations. The CoMFA and CoMSIA studies were performed on these compounds based on three different alignment methods to build 3D-QSAR models. Most of the models showed good *q*^2^ and *r*^2^ _pred_ values and revealed a good response to the test set validation. The CoMFA model generated from the pharmacophore-based method was found to be superior (model 2, *q*^2^ = 0.628, *r*^2^ _pred_ = 0.785) to those obtained from GLIDE docking-based and common substructure based methods. All the CoMSIA models derived from three different alignment methods gave good results, whose *q*^2^ and *r*^2^ _pred_ values were greater than 0.5 and 0.6, respectively. The *q*^2^ value of the best CoMSIA model was only a little larger than that of other models. In view of that, three CoMSIA models were selected for further comparisons and analyses so that more valuable information for the structural requirements can be obtained. From our studies, it was found that the pharmacophore-based alignment produced the best model for CoMFA and the common structure-based alignment for CoMSIA. This indicated that the discovery of the optimal alignment method should depend on the statistical performances of 3D-QSAR models generated from the alignments based on those methods. In addition, suitable alignment methods for CoMFA and CoMSIA studies might be different. The comparative studies among the best CoMFA and the CoMSIA models were also demonstrated in the crystallographic environment of PLK1 and high consistency was found in steric, electrostatic and hydrogen bond donor fields. Furthermore, the contours of CoMSIA model 6 were compared with the structure-based pharmacophore model and the key factors related to binding affinity were reconfirmed. These satisfactory insights identified in the present study can be utilized to design and predict new potent compounds as PLK1 inhibitor candidates, and to discover compounds with novel scaffolds that can act as PLK1 inhibitors via similar mechanisms.

## Figures and Tables

**Table 1:** Structures of 73 compounds.

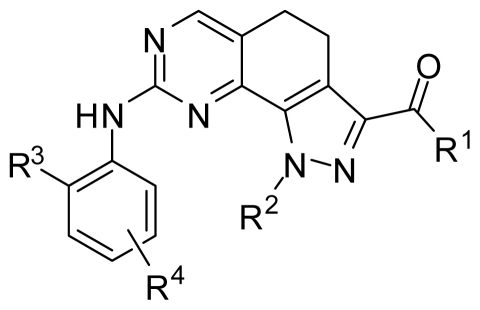				
Compound	R^1^	R^2^	R^3^	R^4^
1 *	NHMe	Me	H	H
2 *	NHcyclopropyl	Me	H	H
3	NHcyclopentyl	Me	H	H
4	NHPh	Me	H	H
5	NH_2_	Me	CF_3_	H
6	NH_2_	Ph	H	H
7	NH_2_	*i*-Pr	H	H
8	NH_2_	1-methylpiperidine-4-yl	H	H
9	NH_2_	2-(piperidin-1-yl)-ethyl	H	H
10*	NH_2_	Me	H	*m*-CF_3_
11	NH_2_	Me	H	*p*-CF_3_
12	NH_2_	Me	Ac	H
13	NH_2_	Me	H	*m*-Ac
14	NH_2_	Me	OMe	H
15	NH_2_	Me	H	*m*-OMe
16	NH_2_	Me	H	*p*-OMe
17	NH_2_	Me	NO_2_	H
18	NH_2_	Me	H	*m*-NO_2_
19	NH_2_	Me	Me	H
20 *	NH_2_	Me	NHMe	H
21	NH_2_	Me	*i*-Pr	H
22 *	NH_2_	Me	COOMe	H
23 *	NH_2_	Me	CONH_2_	H
24	NH_2_	Me	SO_2_NH_2_	H
25	NH_2_	Me	Ph	H
26 *	NH_2_	Me	OPh	H
27	NH_2_	Me	benzyl	H
28 *	NH_2_	Me	NHPh	H
29	NH_2_	Me	benzoyl	H
30 *	NH_2_	Me	SPh	H
31	NH_2_	Me	Ac	3′-(4-methyl-piperazin-1-yl)
32 *	NH_2_	Me	Ac	4′-(4-methyl-piperazin-1-yl)
33	NH_2_	Me	Ac	5′-(4-methyl-piperazin-1-yl)
34	NH_2_	Me	OMe	4′-(4-methyl-piperazin-1-yl)
35 *	NH_2_	Me	OMe	5′-(4-methyl-piperazin-1-yl)
36	OEt	Me	H	H
37	NH_2_	Me	NH_2_	H
38	NH_2_	Me	NHAc	H
39	NH_2_	Me	OCF_3_	H
40	NH_2_	Me	OCF_3_	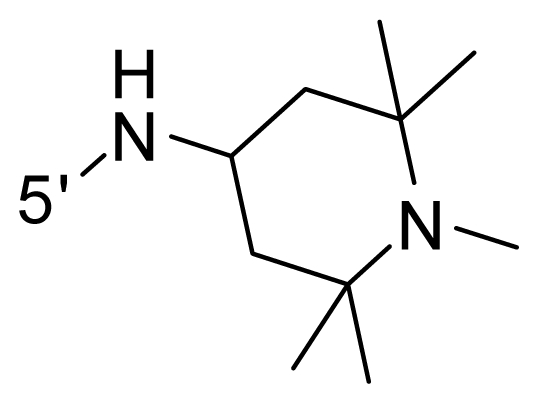
41 *	NH_2_	Me	OCF_3_	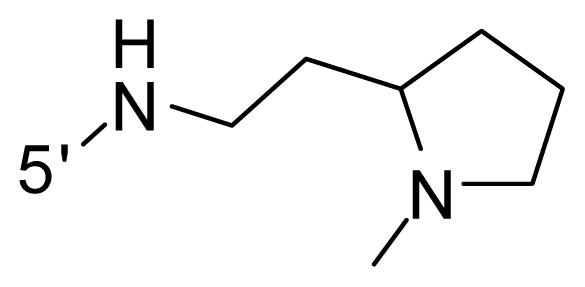
42	NH_2_	Me	OCF_3_	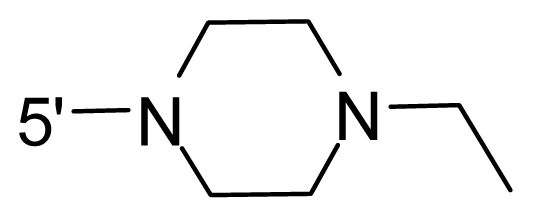
43	NH_2_	Me	OCF_3_	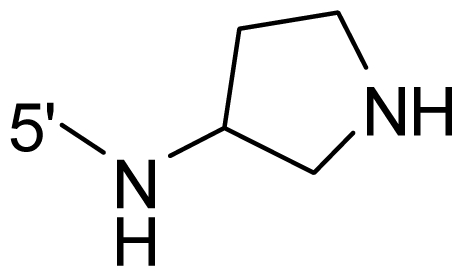
44	NH_2_	Me	OCF_3_	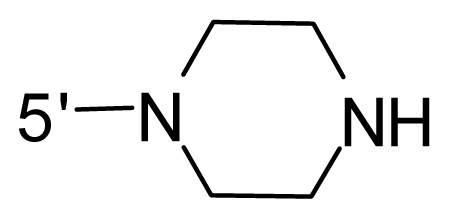
45	NH_2_	Me	OCF_3_	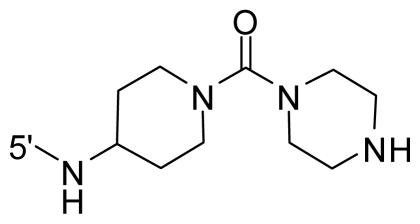
46 *	NH_2_	Trityl	OCF_3_	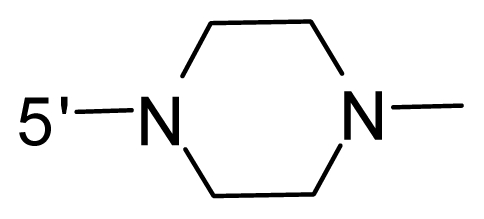
47	NH_2_	H	OCF_3_	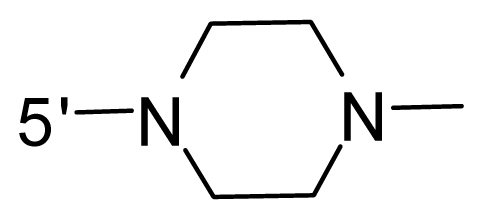
48 *	NH_2_	2-Fluoro-ethyl	OCF_3_	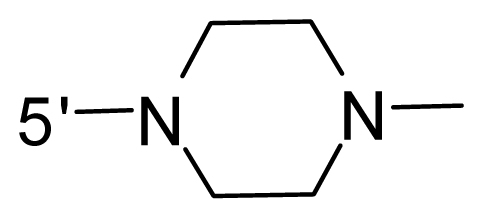
49	NH_2_	Ethyl	OCF_3_	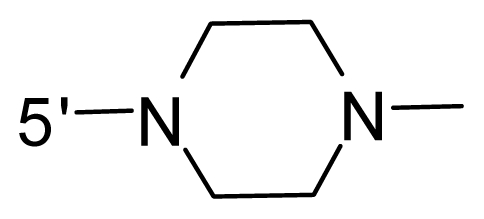
50 *	NH_2_	2-Methoxy-ethyl	OCF_3_	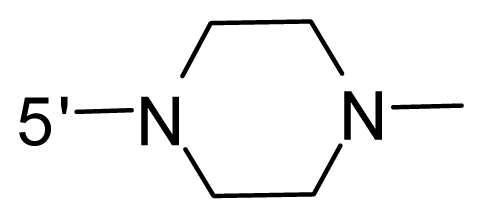
51	NH_2_	2-Chloro-ethyl	OCF_3_	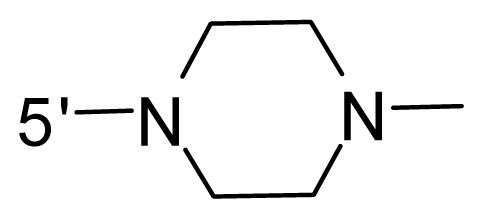
52	NH_2_	Vinyl	OCF_3_	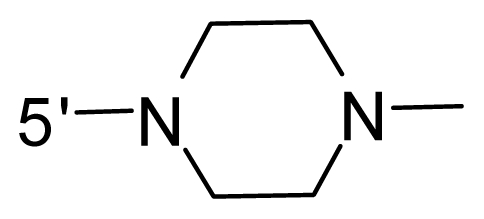
53 *	NH_2_	Me	OCF_3_	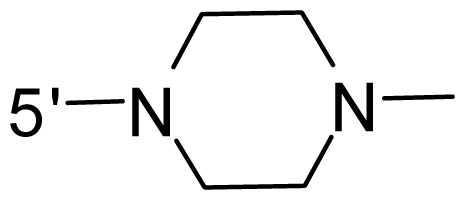
54	NH_2_	Me	OCF_3_	5′-NH_2_
55	NH_2_	Me	OCF_3_	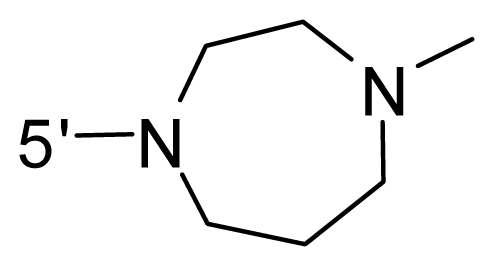
56 *	NH_2_	Me	OCF_3_	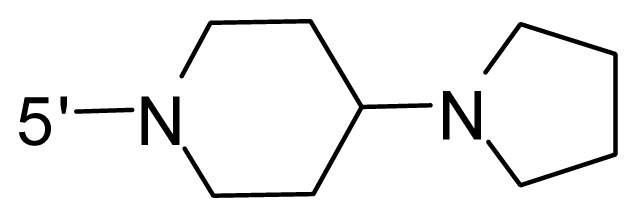
57	NH_2_	Me	OCF_3_	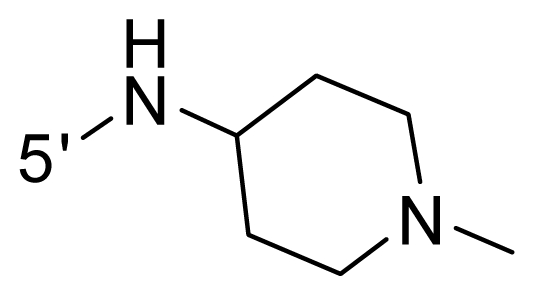
58	NH_2_	Me	OCF_3_	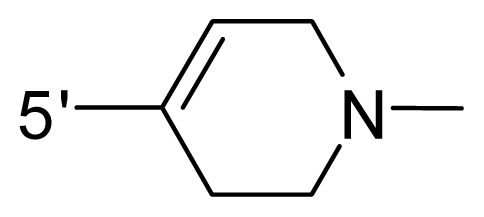
59	NH_2_	Me	OCF_3_	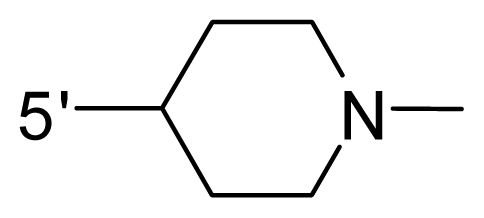
60	NH_2_	Me	OCF_3_	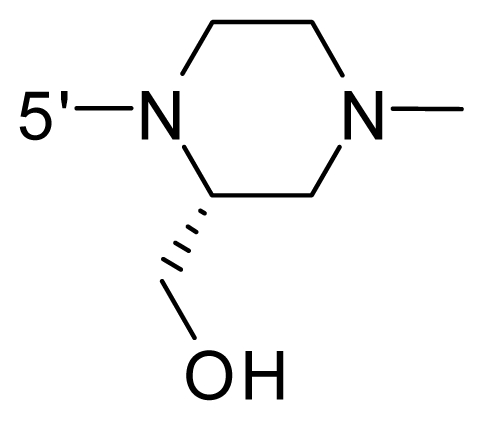
61	NH_2_	Me	OCF_3_	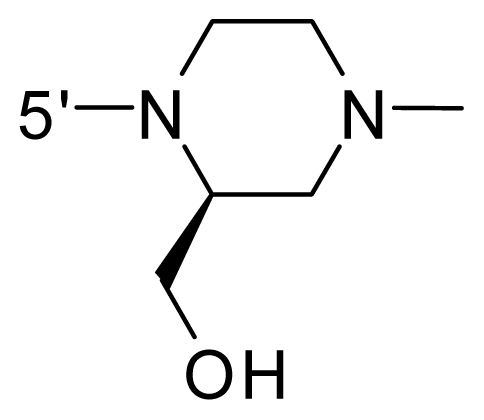
62 *	NH_2_	Me	OCF_3_	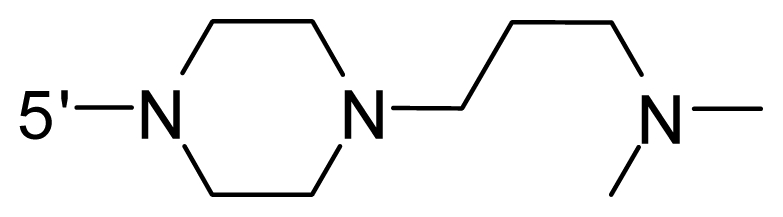
63	NH_2_	Me	OCF_3_	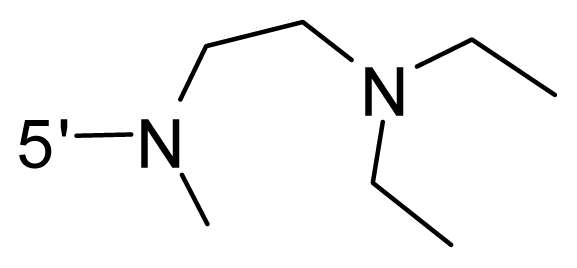
64 *	NH_2_	Me	OCF_3_	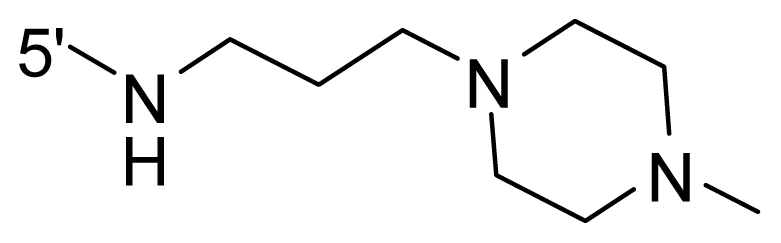
65	NH_2_	Me	OCF_3_	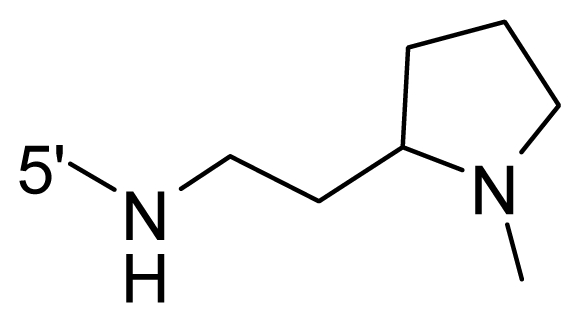
66 *	NH_2_	Me	OCF_3_	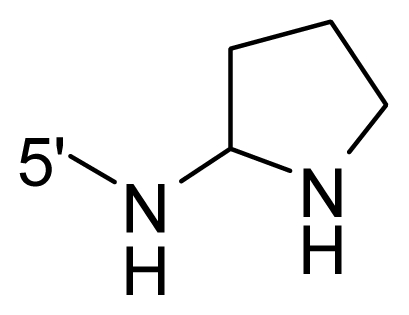
67	NH_2_	Me	OCF_3_	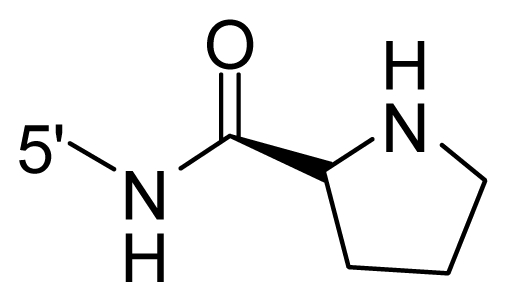
68	NH_2_	Me	OCF_3_	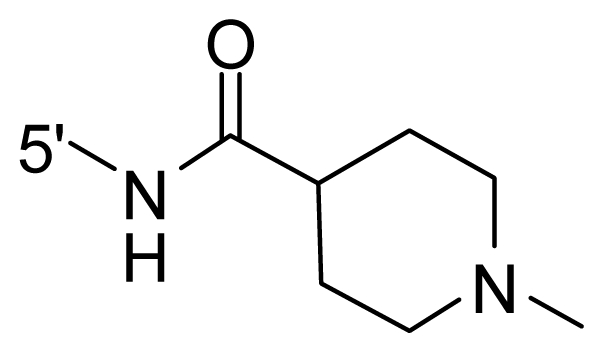
69	NH_2_	−(CH_2_)_3_–N–(CH_3_)_2_	OCF_3_	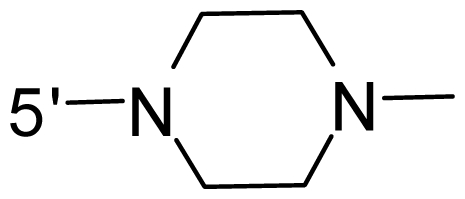
70 *	NH_2_	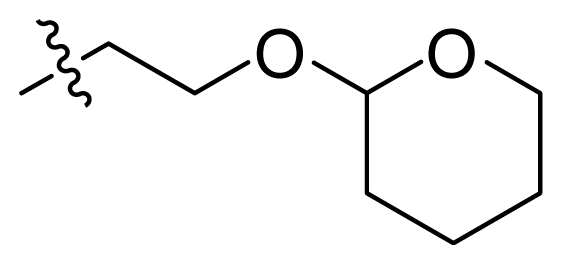	OCF_3_	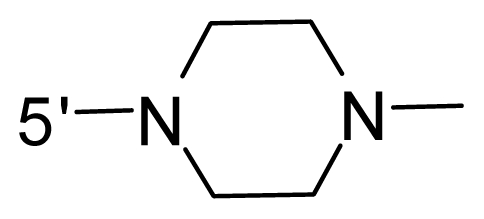
71	NH_2_	− (CH_2_)_2_–NH_2_	OCF_3_	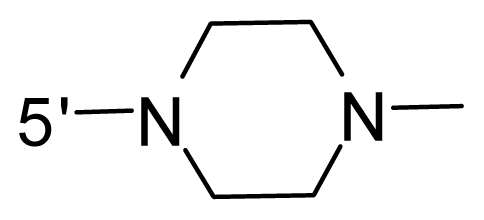
72	NH_2_	− (CH_2_)_3_–NH_2_	OCF_3_	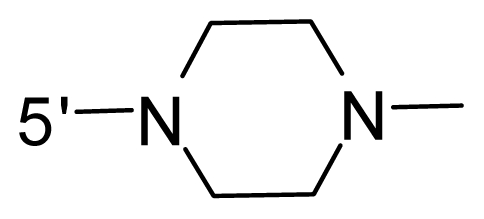
73	NH_2_	− (CH_2_)_2_–OH	OCF_3_	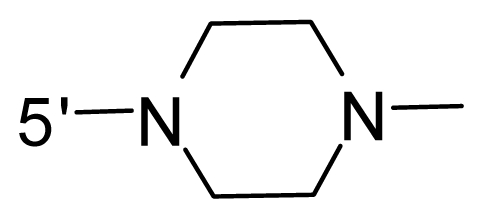

* Compounds in test set.

**Table 2:** Statistics summary of CoMFA and CoMSIA models.

Alignment Method	CoMFA Model			CoMSIA Model		

	GD a	PH b	CS c	GD	PH	CS
No.	1	2	3	4	5	6
*q*^2^	0.283	0.628	0.578	0.574	0.532	0.588
*r*^2^	0.42	0.941	0.867	0.97	0.859	0.834
SEE d	0.818	0.268	0.404	0.198	0.411	0.447
*F*^e^	36.929	192.635	78.313	207.094	99.622	81.965
ONC f	1	4	4	7	3	3
Field analysis						
Steric	0.663	0.619	0.698	0.177	0.224	0.263
Electrostatic	0.337	0.381	0.618	0.52	0.407	0.459
Hydrophobic	-	-	-	-	-	-
H-bond donor	-	-	-	0.303	0.369	0.277
H-bond acceptor	-	-	-	-	-	-
Test set						
*r*^2^_pred_^g^	0.405	0.785	0.752	0.605	0.695	0.749

a Glide docking;

b Pharmacophore;

c Common substructure;

d Standard estimated error;

e Fisher value;

f Optimal number of components;

g Predictive correlation coefficient for test set.

**Table 3:** The experimental pIC_50_, predicted pIC_50_ and residual values of all compounds derived from the CoMFA models.

Compound	pIC_50_	Model 1		Model 2		Model 3	

		Prediction	Residue	Prediction	Residue	Prediction	Residue
1 *	5.375	5.848	−0.473	5.227	0.148	5.380	−0.005
2 *	5.000	5.934	−0.934	5.406	−0.406	5.063	−0.063
3	5.000	5.824	−0.824	5.228	−0.228	4.778	0.222
4	5.000	5.724	−0.724	4.795	0.205	5.298	−0.298
5	6.365	6.544	−0.180	6.382	−0.018	6.282	0.082
6	5.000	6.101	−1.101	5.591	−0.591	5.653	−0.653
7	6.367	6.074	0.293	5.935	0.432	5.765	0.602
8	5.000	6.394	−1.394	5.096	−0.096	4.798	0.202
9	5.000	6.543	−1.543	4.966	0.034	5.060	−0.060
10 *	7.292	6.249	1.043	7.358	−0.066	6.423	0.869
11	6.060	5.547	0.513	6.153	−0.093	6.165	−0.106
12	6.461	6.536	−0.075	6.401	0.060	6.286	0.175
13	7.000	5.914	1.086	7.105	−0.105	6.575	0.425
14	7.377	6.378	0.999	7.359	0.018	7.120	0.257
15	6.870	6.068	0.802	6.798	0.072	6.649	0.221
16	6.592	5.970	0.622	6.402	0.190	6.886	−0.294
17	6.312	6.354	−0.042	6.395	−0.083	6.125	0.187
18	5.000	5.892	−0.892	5.074	−0.074	6.189	−1.189
19	7.824	6.040	1.784	7.420	0.404	6.858	0.966
20 *	6.959	6.260	0.699	6.240	0.719	7.193	−0.234
21	6.438	6.216	0.222	6.485	−0.047	6.749	−0.311
22 *	5.952	6.490	−0.538	6.844	−0.892	6.745	−0.793
23 *	5.683	6.250	−0.567	5.481	0.202	6.781	−1.098
24	5.428	6.150	−0.722	5.564	−0.136	6.110	−0.682
25	5.806	6.316	−0.511	6.198	−0.393	6.044	−0.238
26 *	6.556	6.624	−0.068	6.279	0.277	6.216	0.340
27	6.026	6.216	−0.191	6.005	0.021	6.035	−0.010
28 *	6.023	6.609	−0.586	6.150	−0.127	6.212	−0.189
29	5.706	6.380	−0.674	5.686	0.020	5.724	−0.018
30 *	5.692	6.772	−1.080	6.168	−0.476	6.094	−0.402
31	5.688	5.964	−0.276	5.904	−0.216	5.913	−0.225
32 *	6.334	6.768	−0.435	5.746	0.587	7.694	−1.361
33	6.963	7.711	−0.748	7.152	−0.189	6.698	0.265
34	7.398	6.327	1.071	7.573	−0.175	7.330	0.068
35 *	8.155	7.441	0.714	8.503	−0.348	8.226	−0.071
36	5.000	5.874	−0.874	5.070	−0.070	5.195	−0.195
37	6.824	6.153	0.671	6.920	−0.096	6.796	0.028
38	5.598	5.973	−0.375	5.145	0.453	5.588	0.010
39	6.932	6.387	0.545	6.681	0.251	6.716	0.216
40	6.016	7.373	−1.357	6.026	−0.010	5.755	0.261
41 *	6.910	7.384	−0.474	6.320	0.590	7.188	−0.278
42	8.097	7.501	0.596	8.130	−0.033	8.653	−0.556
43	7.114	7.318	−0.204	7.163	−0.050	7.056	0.058
44	8.699	7.428	1.271	8.567	0.132	8.275	0.424
45	7.456	7.522	−0.066	7.163	0.293	7.905	−0.449
46 *	5.000	7.379	−2.379	5.528	−0.528	4.123	0.877
47	8.398	7.156	1.242	8.361	0.037	8.363	0.035
48 *	8.398	7.276	1.122	7.868	0.530	8.064	0.334
49	8.222	7.609	0.613	7.959	0.263	8.069	0.153
50 *	7.824	7.300	0.524	7.527	0.297	7.675	0.149
51	8.155	7.316	0.839	8.152	0.003	7.918	0.237
52	8.222	7.518	0.704	8.339	−0.117	8.073	0.149
53 *	8.523	7.555	0.968	8.221	0.302	8.579	−0.056
54	7.222	6.432	0.790	7.500	−0.278	7.182	0.040
55	7.602	7.416	0.186	7.967	−0.365	7.685	−0.083
56 *	7.482	7.453	0.028	6.750	0.732	8.656	−1.175
57	6.699	7.505	−0.806	7.303	−0.604	6.568	0.131
58	8.523	7.497	1.026	8.082	0.441	8.499	0.024
59	8.222	7.333	0.889	8.070	0.152	8.018	0.204
60	6.813	7.516	−0.704	6.580	0.233	7.415	−0.603
61	6.697	7.502	−0.805	6.439	0.258	7.358	−0.661
62 *	7.377	7.417	−0.040	7.465	−0.088	7.735	−0.358
63	7.222	7.129	0.093	7.568	−0.346	6.608	0.614
64 *	6.975	7.354	−0.379	6.220	0.755	7.077	−0.102
65	6.827	7.660	−0.833	6.591	0.236	7.034	−0.207
66 *	7.081	7.358	−0.277	6.546	0.535	6.968	0.113
67	7.357	7.555	−0.199	7.285	0.071	6.833	0.523
68	6.650	7.634	−0.984	6.181	0.469	6.117	0.533
69	6.554	7.291	−0.737	6.763	−0.209	6.665	−0.111
70 *	7.367	7.521	−0.155	6.587	0.780	7.756	−0.390
71	7.482	7.562	−0.081	8.013	−0.532	7.765	−0.284
72	7.284	7.647	−0.363	7.332	−0.048	7.703	−0.419
73	8.699	7.541	1.158	8.292	0.407	8.235	0.464

* Test set compounds.

**Table 4:** The experimental pIC_50_, predicted pIC_50_ and residual values of all compounds derived from the CoMSIA models.

Compound	pIC_50_	Model 4		Model 5		Model 6	

		Prediction	Residue	Prediction	Residue	Prediction	Residue
1 *	5.375	5.171	0.204	5.208	0.167	4.833	0.542
2 *	5.000	5.524	−0.524	5.168	−0.168	4.781	0.219
3	5.000	5.037	−0.037	5.159	−0.159	4.620	0.380
4	5.000	5.099	−0.099	5.024	−0.024	5.314	−0.314
5	6.365	6.248	0.116	6.307	0.057	6.225	0.140
6	5.000	4.967	0.033	5.446	−0.446	5.907	−0.907
7	6.367	6.230	0.137	5.931	0.436	5.911	0.456
8	5.000	5.102	−0.102	5.189	−0.189	4.950	0.050
9	5.000	5.239	−0.239	5.014	−0.014	5.194	−0.194
10 *	7.292	6.092	1.200	6.508	0.784	6.233	1.059
11	6.060	6.063	−0.003	5.964	0.095	6.148	−0.088
12	6.461	6.453	0.008	6.401	0.060	6.052	0.409
13	7.000	7.012	−0.012	6.302	0.698	6.818	0.182
14	7.377	7.070	0.307	7.380	−0.003	6.911	0.466
15	6.870	7.040	−0.170	6.452	0.418	6.517	0.353
16	6.592	6.660	−0.068	6.576	0.016	6.926	−0.334
17	6.312	6.395	−0.083	6.653	−0.341	6.169	0.143
18	5.000	5.062	−0.062	5.449	−0.449	5.846	−0.846
19	7.824	7.011	0.813	6.951	0.873	6.576	1.248
20 *	6.959	6.528	0.431	5.916	1.043	6.666	0.293
21	6.438	6.699	−0.261	6.742	−0.304	6.645	−0.207
22 *	5.952	6.461	−0.509	6.993	−1.041	6.700	−0.748
23 *	5.683	5.490	0.193	5.590	0.093	6.630	−0.947
24	5.428	5.327	0.101	5.574	−0.146	5.521	−0.093
25	5.806	5.896	−0.090	6.583	−0.778	6.265	−0.459
26 *	6.556	5.894	0.662	6.111	0.445	6.740	−0.184
27	6.026	5.814	0.212	6.347	−0.322	6.295	−0.270
28 *	6.023	5.744	0.279	6.477	−0.454	6.749	−0.726
29	5.706	5.708	−0.002	5.322	0.384	6.148	−0.442
30 *	5.692	6.086	−0.394	6.111	−0.419	6.328	−0.636
31	5.688	5.601	0.087	6.117	−0.429	6.026	−0.338
32 *	6.334	8.044	−1.711	6.289	0.045	7.253	−0.920
33	6.963	6.937	0.026	6.742	0.221	6.561	0.402
34	7.398	7.349	0.049	7.880	−0.482	7.183	0.215
35 *	8.155	7.191	0.964	7.979	0.176	8.207	−0.052
36	5.000	5.047	−0.047	4.871	0.129	5.260	−0.260
37	6.824	6.803	0.021	6.603	0.221	7.167	−0.343
38	5.598	5.651	−0.053	5.731	−0.133	5.382	0.216
39	6.932	7.623	−0.691	6.973	−0.041	6.569	0.363
40	6.016	6.033	−0.017	6.427	−0.411	5.770	0.246
41 *	6.910	6.012	0.898	6.753	0.157	7.180	−0.270
42	8.097	8.147	−0.050	8.069	0.028	8.308	−0.211
43	7.114	7.115	−0.002	6.944	0.170	6.619	0.495
44	8.699	8.774	−0.075	8.472	0.227	8.358	0.341
45	7.456	7.337	0.119	6.944	0.512	7.701	−0.245
46 *	5.000	5.778	−0.778	5.855	−0.855	4.419	0.581
47	8.398	8.364	0.034	8.644	−0.246	8.514	−0.116
48 *	8.398	8.319	0.079	8.168	0.230	7.910	0.488
49	8.222	8.041	0.181	7.951	0.271	7.944	0.278
50 *	7.824	7.912	−0.088	7.834	−0.010	7.619	0.205
51	8.155	8.241	−0.086	7.889	0.266	7.766	0.389
52	8.222	8.378	−0.156	8.125	0.097	7.931	0.291
53 *	8.523	8.002	0.521	8.126	0.397	8.239	0.284
54	7.222	7.369	−0.147	7.428	−0.206	7.069	0.153
55	7.602	7.615	−0.013	8.039	−0.437	7.530	0.072
56 *	7.482	7.503	−0.022	7.064	0.418	8.246	−0.765
57	6.699	6.573	0.126	6.999	−0.300	6.315	0.384
58	8.523	8.441	0.082	7.866	0.657	8.501	0.022
59	8.222	8.102	0.120	8.128	0.094	7.886	0.336
60	6.813	6.696	0.117	6.928	−0.116	7.765	−0.953
61	6.697	6.888	−0.191	6.960	−0.263	7.513	−0.816
62 *	7.377	7.339	0.038	7.754	−0.377	7.886	−0.509
63	7.222	7.253	−0.031	7.863	−0.641	6.816	0.406
64 *	6.975	6.454	0.521	5.790	1.185	7.407	−0.432
65	6.827	6.790	0.037	6.234	0.593	7.473	−0.646
66 *	7.081	6.289	0.792	6.296	0.785	7.145	−0.064
67	7.357	7.293	0.063	6.725	0.632	6.899	0.458
68	6.650	6.515	0.135	6.238	0.412	6.724	−0.074
69	6.554	6.540	0.014	7.249	−0.695	7.200	−0.646
70 *	7.367	6.809	0.558	6.531	0.836	7.257	0.110
71	7.482	7.571	−0.090	7.993	−0.512	7.728	−0.247
72	7.284	7.357	−0.073	7.314	−0.030	7.624	−0.340
73	8.699	8.680	0.019	7.805	0.894	8.294	0.405

* Test set compounds.
